# Unilateral testicular enlargement in a teenager with Beckwith-Wiedemann syndrome: a case report

**DOI:** 10.1186/s13052-019-0675-1

**Published:** 2019-07-10

**Authors:** Maria Chiara Pellegrin, Alessandro Mauro Spinelli, Gianluca Tornese, Egidio Barbi

**Affiliations:** 1 Institute for Maternal and Child Health, IRCCS Burlo Garofolo, Via dell’Istria 65/1, 34137 Trieste, Italy; 2 University of Udine, Udine, Italy; 3 University of Trieste, Trieste, Italy

**Keywords:** Beckwith-Wiedemann syndrome, Testis, Lateralized overgrowth, Puberty

## Abstract

**Background:**

Beckwith-Wiedemann syndrome (BWS) is a rare congenital overgrowth disorder. A major feature is lateralized overgrowth, which can variably involve a single body district up to the entire hemisome. Visceral asymmetrical involvement has been observed, commonly represented by enlargement of one kidney or adrenal gland, rather than one gonad.

**Case presentation:**

We report the case of a pubertal boy affected by BWS, who developed a progressive testicular enlargement, ipsilateral to the pre-existing external body overgrowth. Asymptomatic unilateral testis enlargement started after regular pubertal onset and worsened over time, without any associated pathological findings in a long-term follow-up. Since biopsy is not indicated in case of benign macro-orchidism, we hypothesize that this asymmetric enlargement could be an expression of visceral lateralized overgrowth in BWS.

**Conclusions:**

At the best of our knowledge, this is the first detailed report of unilateral testicular overgrowth in BWS. We revised common causes of painless unilateral scrotal masses in the pediatric age. Considering both the overall frequency of neoplasia and the malignancies predisposition in BWS, a testicular cancer should be carefully ruled out through a close follow-up, before stating a benign condition. A normal ultrasound pattern, together with normal serum hormonal levels and negative tumor markers, make testicular neoplasms highly unlikely.

## Background

Beckwith-Wiedemann syndrome (BWS) is a congenital overgrowth disorder with heterogeneous genetic background and variable phenotypic expression, more recently defined as Beckwith-Wiedemann Spectrum (BWSp) [1]. The condition usually results from dysregulation of the chromosome 11p15 imprinted region and involves overgrowth in multiple tissues, often in a mosaic pattern. It is a rare disease, with an estimated prevalence of 1 affected child per 10,340 live births, most commonly diagnosed in the neonatal period or in early childhood [1]. According to the recent Consensus Statement, a cardinal feature of this condition is lateralized overgrowth (previously known as hemihypertrophy or hemihyperplasia), defined as a significant increase in the length and/or girth of most or all of one side of the body compared with its contralateral side in addition to a chromosome 11p15 molecular anomaly [1, 2]. It might occur in all molecular subtypes of BWSp, but most commonly in patients with paternal uniparental disomy (UPD) for chromosome 11p15 [1]. The overgrowth occurs in the absence of a recognizable pattern of major or minor malformations, dysplasias, or morphologic variants and may involve an entire hemisome, a single limb, one side of the face or combinations thereof (it can be present even in different parts of the body that differ in laterality). The asymmetry may become more pronounced or less noticeable with age [3]. Even if asymmetry primarily concerns external body extremities, overgrowth of an organ may or may not be present [2]. Visceromegaly is a possible associated finding [3], often detectable in the prenatal ultrasonography [1]. Beside enlargement of a single organ (usually liver, spleen, pancreas), patients affected by BWS may present asymmetrical organomegaly of one kidney, adrenal gland, testis or ovary [3]. Both external body and visceral tissues overgrowth represents a risk factor for cancer development [1–3].

## Case presentation

A boy affected by BWS has been followed in our Pediatric Endocrinology Unit to monitor growth and pubertal development. Given the neonatal findings of macrosomia (birthweight >2SDS), transient hyperinsulinemic hypoglycemia and umbilical ‘hernia permagna’ (expression of an anterior abdominal wall defect), a diagnosis of BWS was hypothesized and further confirmed by molecular analysis, that revealed a mosaic paternal isodisomy of chromosome 11p15. Family history was unremarkable. Over the years, linear growth assessed on the 90th centile, with an increasingly evident right-sided overgrowth of the trunk and limbs (leg length discrepancy of 2 cm, managed with shoe-lifts in order to avoid pelvic tilt and scoliosis). No embryonal tumors occurred. Pubertal development started regularly at 11 years of age with bilateral testicular volume of 5 ml, associated with phallus enlargement. At the age of 13 years, a slight testicular asymmetry was noticed. At 14 years testicular asymmetry was prominent: clinically estimated testicular volume was 18 ml on the right and 12 ml on the left side. The bigger testis appeared of normal consistency, without intra or paratesticular nodular masses. Scrotal transillumination was opaque. The boy was asymptomatic and denied traumas. Growth rate velocity was consistent with normal pubertal spurt.

**Fig. 1:**
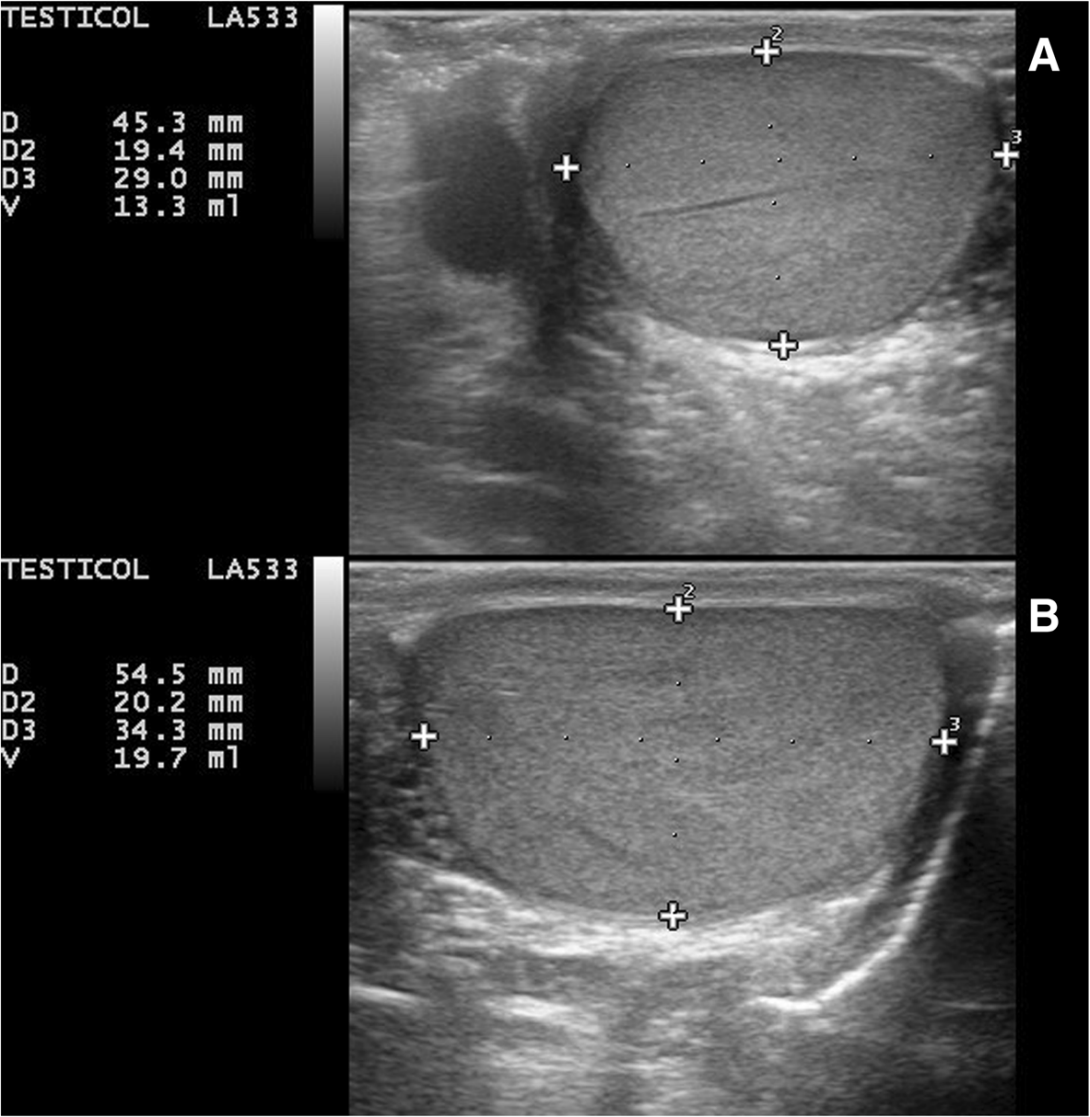
Testicular ultrasound of our 15-years-old patient showed normal sonographic appearance of both testes and epididymides and confirmed volumetric asymmetry (right testis measuring 54.5 x 20.2 x 34.3 mm with an estimated volume of 19.7 ml, left testis measuring 46.2 x 18.6 x 29.1 mm with an estimated volume of 13.3 ml).

Testicular ultrasound confirmed the clinical asymmetry (right testis measuring 46 × 31 × 24 mm and left testis measuring 35 × 23 × 14 mm, with an estimated volume of 18 ml and 12 ml, respectively) and showed a regular echostructure of both testes and epididymides, excluding focal lesions. Abdomen ultrasound was negative for alterations. Laboratory studies, including complete blood count, lactic dehydrogenase, serum tumor markers (bHCG, aFP, CEA), testicular, adrenal and thyroid hormones were repeatedly normal. In the absence of clinical signs of malignancy, a watch and wait approach was preferred to a biopsy. Asymmetry slightly increased during the follow-up: at 15 years of age, right testis measured 54.5 × 20.2 × 34.3 mm by ultrasound with an estimated volume of 19.7 ml and left testis measured 46.2 × 18.6 × 29.1 mm with an estimated volume of 13.3 ml (Fig. 1), with a difference between right and left gonad that remained fairly stable. Meanwhile, leg length discrepancy maintained a 2 cm difference. Clinical and laboratory markers of neoplasia, repeated every six months, were always negative. In the absence of malignancy or other tissue focal malformations in a 4 years follow-up, our final diagnosis was visceral benign asymmetry.

## Discussion and conclusions

Different urologic manifestations have been described in BWS, most commonly renal abnormalities [4, 5]. Other urologic manifestations have been less well described in the literature. Testicular anomalies reported in BWS are illustrated in Table 1. Cryptorchidism represents the most common finding, especially in patients with loss of methylation of imprinting center 2 [5], while gonadoblastoma has been reported as a possible cause of intratesticular neoplasia [4]. Testicular interstitial cell hyperplasia was documented in the first report of this disorder in 1963, while in newborns both bilateral testes enlargement and a Leydig-cell hyperplasia (not associated to increased testicular size) have been reported [4, 6, 7]. There are no specific reports on pubertal age in BWS. Gonads asymmetry has been mentioned together with other more common sites of visceral asymmetrical enlargement, instead the overgrowth of a single testis triggered by puberty has never been reported before.

**Table 1:** Causes of asymptomatic unilateral scrotal masses in the pediatric age and testicular abnormalities in Beckwith-Wiedemann syndrome

Benign conditions - hydrocele, hernia, varicocele, spermatocele/epididymal cyst, sebaceous cyst - benign idiopathic macro-orchidism - supernumerary testis - organized post-traumatic hematocele	
Testicular and paratesticular neoplasms - germ cell tumors (epidermoid cyst, teratoma, germinoma, yalk sac tumor, embryonal carcinoma, mixed germ cell tumor) - gonadal stromal tumors (Leydig or Sertoli cell tumors) - lipoma, fibroma, leiomyoma, lymphangioma, fibrosarcoma, rhabdomyosarcoma - leukemic infiltration, lymphoma, metastasis (nephroblastoma, neuroblastoma) - McCune-Albright syndrome	
Inflammation/infection - epididymo-orchitis (extrapulmonary tuberculosis)	
Compensatory hypertrophy (in the absence of the contralateral testicle)	
Testicular abnormalities in Beckwith-Wiedemann syndrome - cryptorchidism - gonadal interstitial-cell hyperplasia - gonadoblastoma - enlarged testicle - hypoplastic testicle - agenesis of the testicle	

Causes of painless unilateral scrotal masses, originating from testis, epididymis, spermatic cord or scrotal wall [7–10]. Endocrinopathies like adrenal remnants in congenital adrenal hyperplasia, precocious puberty, Leydig cell hyperplasia due to familial testotoxicosis have not been included, as they generally determine synchronous bilateral testicular enlargement. In addition, the reported testicular variances in BWS [4].

Looking at the entire pediatric age group, differential diagnosis of painless unilateral testicular enlargement ranges from benign manifestations to testicular or extra-testicular tumors (see Table 1).

Since tumors represent the major cause of pediatric painless scrotal masses (accounting for more than 70% of cases) [8], this condition is by definition cancerous until proven otherwise [9]. A physical examination does not adequately identify all the masses, hence imaging and biochemical data are critical. Ultrasound is considered the imaging modality of choice in the evaluation of asymmetrical scrotal masses, because it is a noninvasive method with high sensitivity for the detection of testicular neoplasms (nearly 100%, with a negative-predictive value of almost 100%): when testes have homogeneous normal echogenicity, these always corresponds to a benign pathology (supernumerary testis, unilateral testicular hypertrophy, bilateral hypertrophy in BWS) [7–9]. Unilateral testicular hypertrophy (also called idiopathic benign macro-orchidism) is a possible cause of painless testis enlargement in the general population. This is a rare condition (incidence not reported), described as a normal variant in healthy subjects, probably due to Leydig cells hyperplasia and not associated with an underlying pathological process or with abnormalities of contralateral testis [10]. It can occur during childhood or adolescence, bilaterally or unilaterally, suggesting a different receptor response of the testes to gonadotropins and androgens. It can persist or disappear completely over time [9, 10]. Clinical, sonographic and laboratory features of this condition are distinct from the other causes of testicular enlargement (normal ultrasound, negative tumor markers and normal biochemical analysis) and allow a diagnosis without surgical exploration or testicular biopsy [10].

As the history of our patient was negative for cryptorchidism and testicular torsion, compensatory hypertrophy was excluded. On the other hand, by considering that asymmetric overgrowth has initiated at pubertal time and given the oncogenic risk in BWS, especially in case of chromosome 11p15 UPD [1], malignancies have been immediately considered and subsequently excluded through a strict clinical, biochemical and imaging follow-up. Ultrasound confirmed the asymmetry and demonstrated a persistent normal testicular echogenicity, correlating this finding to benign idiophatic macro-orchidism and avoiding a biopsy (a conservative approach is preferred for healthy individuals with this condition).

Without a biopsy, pathological findings cannot be assessed and an accurate final diagnosis cannot be made, but considering genetic predisposition and pre-existing body asymmetry, we cannot exclude that this event was an expression of lateralized visceral overgrowth in BWS.

In conclusion, we report an uncommon case of progressive asymptomatic testis enlargement in an adolescent affected by BWS: it occurred slowly and asymptomatically, within a regular pubertal development and consensually to a pre-existing body asymmetry. In a long term follow-up, a completely normal ultrasound examination together with persistent negative evaluation for tumor markers strongly suggest a benign condition. A conservative approach may be acceptable if a strict follow-up is available, considering that this syndromic disorder is associated with an increased risk of tumors.

## Data Availability

Data sharing not applicable to this article as no datasets were generated or analysed during the current study.

## Abbreviations

aFP: Alpha-fetoprotein
bHCG: Beta-Human chorionic gonadotropin
BWS: Beckwith–Wiedemann syndrome
BWSp: Beckwith-Wiedemann Spectrum
CEA: Carcinoembryonic antigen
UPD: Uniparental disomy
